# Edible Insects for Humans and Animals: Nutritional Composition and an Option for Mitigating Environmental Damage

**DOI:** 10.3390/insects13100944

**Published:** 2022-10-18

**Authors:** Roberto Ordoñez-Araque, Nadine Quishpillo-Miranda, Luis Ramos-Guerrero

**Affiliations:** 1 Escuela de Gastronomía, Universidad de Las Américas (UDLA), Quito 170124, Ecuador; 2 Facultad de Salud y Bienestar, Escuela de Nutrición y Dietética, Universidad Iberoamericana del Ecuador (UNIB.E), Quito 170522, Ecuador; 3 Centro de Investigación de Alimentos (CIAL), Universidad UTE, Quito 170129, Ecuador; 4 Agencia de Regulación y Control Fito y Zoosanitario (AGROCALIDAD), Quito 170518, Ecuador

**Keywords:** edible insects, food security, food safety

## Abstract

**Simple Summary:**

A potential substitute to reduce the greenhouse gas emissions caused by the current food business is edible insects. This is possible since eating insects contributes to a healthy dietary pattern and lowers the use of nonrenewable resources. This study describes the primary nutritional contributions of insects, emphasizing their protein content (20–70%), amount of essential amino acids (46–96%), lipid content (10–50%)—which includes saturated and unsaturated fatty acids—fiber content (8.5–27%), and contribution of minerals (primarily calcium, copper, manganese, and zinc) and vitamins (mainly B complex vitamins). The concentrations of nutrients can vary considerably according to the species of insect and its stage of development. It can be concluded that the consumption of insects in humans and animals can reduce the environmental impact on the planet and provide a food source that meets all the nutrients necessary for a daily intake.

**Abstract:**

Every day, there is an increase in environmental damage on the planet regarding human action. One of the causes is food production. Edible insects are presented as an option to mitigate the environmental damage generated by the production of conventional food for humans and animals. The objective of this study was to investigate the main nutritional aspects of insects and how they can provide a nutritional and sustainable alternative to the planet. As the main results, the nine orders of insects that are most consumed on the planet are presented: Blattodea, Coleoptera, Diptera, Hemiptera, Hymenoptera, Isoptera, Lepidoptera, Odonata, and Orthoptera. Their main macro- and micronutrient aspects as well as their bioavailable and bioaccessibility proteins and essential amino acids, monounsaturated fatty acids, minerals, vitamins, and fiber (chitin) are also explored. Additionally, some of the species that are used for animal food processing and the possible risks that insects can present when used as food are discussed. With this, edible insects are established as a real option to mitigate climate change being an important nutritional source for the development of food for humans and animals.

## 1. Introduction

In the last century, the population has experienced exponential growth. There are now almost seven times more people in the world than in the eighteenth century, mainly because most developing countries are going through the second stage of the demographic transition [1]. According to the United Nations, population growth has been observed every decade. Since the Second World War (1939–1945), the planet has experienced an increase of 1 billion people every 12 to 15 years. It is estimated that, since 1950, the population has grown by 250%, from 2.6 billion to 8 billion today. Projections indicate that, by the year 2050, the world population will be between 9.7 and 10 billion people [2,3]. Currently, this population growth represents a challenge for the planet because the demand for food of animal and vegetable origin increases year by year, and this leads to a progressive increase in greenhouse gas emissions along with the overuse of water and land resources. All this causes considerable changes in the environment and contributes to climate change [4].

The need for food is intensified by population growth. Livestock production is one of the sectors that contributes most to climate change, with the significant contribution of methane (CH_4_), carbon dioxide (CO_2_), and nitrous oxide (N_2_O) to the atmosphere. These greenhouse gases cause an increase in the temperature of the planet, thereby causing changes in ecosystems [5]. Pig and chicken production also has a significant environmental impact (less than intensive livestock production), as these animals contribute significant amounts of nitrogen (N) and phosphorus (P) into the environment [6]. Animal protein production generates a considerable environmental impact depending on the species and the production system. In general, animals, throughout their life until they reach the consumer’s table, need large extensions of land for their breeding, along with a large amount of food resources (these also generate an environmental impact from cultivation to processing) and water, fossil fuels, cleaning agents, and packaging [7].

Another cause of climate change is agricultural production, as it has displaced and replaced natural ecosystems in every country on the planet. It is currently the greatest threat to the loss of flora and fauna on the earth’s surface. Agriculture is a major source of greenhouse gases, especially with carbon emissions [8]. It should also be noted that agriculture consumes around 70% of the fresh water of the planet. In addition to these problems, the growing annual demand for food of animal origin has led to the continued expansion of agricultural areas. At present, approximately 80% of the planet’s agricultural land is used to produce animal feed and livestock grazing. It should also be noted that intensive livestock farming is expanding by displacing agricultural areas that were intended for crops or forest areas, causing a loss of natural resources that will lead to changes in the environmental system [8,9].

After analyzing the environmental problems that the planet has experimented because of agriculture and animal production, it is necessary to find a viable and nutritious solution that mitigates, to some extent, the pollution generated by traditional food production. This is where insects come in. It is estimated that there are some 750,000 species, of which 2000 are currently considered edible for both humans and animals [4]. They can be considered a nutritional alternative in the daily diet of people and animals because of the macro- and micronutrients they possess. In general, they have all amino acids which are essential for humans, a protein content that is between 40 to 65% (dry matter) depending on the species, mono- and polyunsaturated fatty acids, vitamins, and minerals. The WHO has established that the consumption of insects could cover the daily needs of nutrients if they are consumed with other foods that contribute to a healthy and balanced diet [10,11]. One of the main advantages of insects is that their feed has the capacity to generate considerably fewer greenhouse gas emissions, they require less food and water consumption, and require negligible land space compared to traditional crops [12].

That there are changes in habits in the adult population is a complicated problem which is exemplified in the dietary habits of people. Introducing edible insects or insect-based foods can be complex for the human paradigm (for ethical, religious, social, cultural, and psychological reasons) [13]. A first step to reduce the environmental footprint that indirectly generates the consumption of traditional animal-based foods may be to substitute plant-based feeds with insect-based foods. These can have the same nutritional characteristics as conventional animal feed, but at the time of their development and production, they will generate a lower impact on the environment. This will also introduce the idea among consumers that insects can be a viable alternative to replace animal-based food [13].

This review aims to analyze the nutritional value of edible insects, their possible use as human and animal food instead of traditional food, and review how starting new eating habits can improve the environment on the planet.

## 2. Edible Insects in Humans and Animals

As indicated, it is estimated that there are more than 2000 insect species viable to be consumed around the world, but only a small fraction have been analyzed for their nutritional composition. It is generally known that insects are an important source of mainly proteins and lipids, but the amounts present in each species may vary according to several factors such as developmental stage, rearing conditions, feeding, ecological aspects, and industrialization [14]. In general, nine orders of insects are the most consumed in the world: Blattodea (cockroaches), Coleoptera (beetles), Diptera (flies), Hemiptera (true bugs), Hymenoptera (ants, bees, and wasps), Isoptera (termites), Lepidoptera (caterpillars, butterflies, and moths), Odonata (dragonflies and damselflies), and Orthopera (crickets, grasshoppers, and locusts); in Africa, the order of Mantodea (mantis) is also included which has a consumption of less than 1% in the continent [15,16,17].

The consumption of insects has been carried out throughout the history of mankind, mainly in Asia, Australia, America, and the African continent, while in Europe its consumption is referred to at the beginning of the 20th century [18]. There are some species of edible insects that are commonly commercialized in several countries; the main ones are: yellow meal worm (*Tenebrio molitor* L.), house cricket (*Acheta domesticus* L.), superworm (*Zophobas morio* Fabricius), domesticated silkworm (*Bombys mori* L.), lesser mealworm (*Alphitobius diaperinus* Panzer), mopane caterpillar (*Imbrasia belina*), roach (*Blaptica dubia* Serville), and African palm weevil (*Rhynchoporus phoenicis*) [17,19]. The use of insects in commercial feed formulations represents a possible way to convert animal diets into more sustainable systems, as insects can be an interesting source of protein compared to current commonly used sources [20]. The latest trends in the use of insects for animal diets show that the market for edible insects for animal feed is expected to expand to USD 2.386 billion by the end of 2029 [3].

It should be considered that insects do not necessarily need to be processed in order to be used as feed for chickens and fish; free-range chickens often eat insects naturally. Processed insect meal can be used in feed rations for pigs and cows, replacing expensive protein-rich ingredients such as fishmeal or soybean meal [21]. Insect meal is the most widely produced product, but there has also been an increase in the production of defatted insect meal, which is characterized by a higher protein content (around 60% on a dry basis). Another product that has increased its production in recent years is insect oils, which have a high concentration of lauric, oleic, palmitic, and linoleic acid. Dehydrated and live larvae are also produced [22,23].

The use of insects for animal feed has been studied during the last few years. They have been included in feed at different stages of development: eggs, larvae, pupae, and adults. They have even been fed alive. This is important since not all insects have an ideal nutritional composition during all life stages [4]. To develop a diet balanced in macro- and micronutrients, the nutritional requirements of the animal species must be analyzed in order to evaluate which type of insect and processing may be the most suitable. For this purpose, different technological processes are applied where proteins are concentrated. For example, the lipid fraction is regulated or the amount of chitin and chitosan is increased [24]. Among the predominant species that have been used for animal feed are: yellow meal worm, house crickets, black soldier fly larvae (*Hermetia illucens* L.), superworm, termite (*Macrotermes nigeriensis*), Tukestan cockroaches (*Blatta Lateralis*), and greater wax moth larvae (*Galleria mellonella* L.) [22,24].

Developing insect-based animal feed will represent a considerable decrease in costs for producers with similar or superior results in obtaining animal protein. The production of insect meal can be a protein alternative to partially replace commercial meal intended for animals raised for human consumption; research has been generated where the use of housefly larvae, yellow mealworm, and black soldier fly larvae meal has been tested instead of fish, soybean, and corn meal with the same results in weight and feed efficiency of chickens. Results have also shown that animals achieve weight as a result of an increase in daily feed. Similarly, it should be noted that some insect species can improve the taste of the meat of animals; for example, in the Philippines it has been observed that consumers have a preference for the taste of chickens that have been fed with grasshoppers than with traditional feed [25].

The use of insects in the feeding of egg-producing poultry can also be an option. By substituting fish meal with mealworm meal, an increase in egg production was observed. Traditionally, small farms in Asia and Africa have used insects as fish feed. It has been determined that the use of black soldier fly meal, mealworms, and silkworm pupa can replace the traditional feed without any adverse effect on the animals or alteration in taste, odor, or texture. It is necessary to consider the requirement of omega-3 fatty acids for the development of some species of fish. For this reason, these acids must be provided in the diet of the insects so that they are present in a considerable concentration [24,25]. Studies have also been developed to determine the inclusion of insects in pig diets. Replacing diets with soybean meal and fish meal in the feed of nursery pigs with black soldier fly larvae, which were reared on cattle manure and cricket meal (*Teleogryllus testaceus*), have been observed, demonstrating that they can be an effective alternative in the nutritional aspect and are more palatable for the animal. Finally, it is important to point out that insects are not only a great alternative because of the nutrients they possess and the possible reduction in environmental impact if they are processed more commonly; they also have a higher edible fraction (better feed conversion efficiency) when compared to animal protein. This is due to the fact that they lack several components in their bodies that have no value as food for human beings (for example: bones, cartilage, skin, hair, etc.) [24].

## 3. Nutritional Value

### 3.1. Nutritional Value of Insects

In general, edible insects are a great food source of nutrients for humans and animals. The use of insects for animal feed was first reported in 1919, while their use on a larger scale was from 1960 to 1970. A summary of the most representative nutrients in the different orders of insects is seen in Table 1. It is emphasized that the values can be variable in each order since the studies where their nutritional value was determined were conducted at different stages in the development of each species (the different studies analyzed their nutritional value at relevant stages for the consumption of each insect), with species requiring different types of habitat and feeding, and have been analyzed with different instrumental techniques.

#### 3.1.1. Energy Value

The fatty acid content will represent the main source of energy value. It should be mentioned that this content is in regard to the whole insect, since at the industrial level, there are flour forms in which the energy value is well below normal ranges. Insects, depending on many factors, will have an energy content between 200 to 700 kcal/100 g (dry basis), with the majority having an average value between 400 to 500 kcal/100 g. Several species of flies (Diptera) have a low fat content and their energy value oscillates between 200 kcal/100 g. In the order Lepidoptera, we can find species with high values that can exceed 700 kcal/100 g, as is the case of the caterpillar *Phasus triangularis* with 776.9 kcal/100 g. In general, we find many edible insects such as crickets, grasshoppers, locusts, and caterpillars in the range of 400 to 500 kcal/100 g, varying according to the species and their nutrient content. No studies have been found where the energy value of insects of the orders Isoptera and Blattodea were reported, although it can be deduced that their energy value will be similar to the rest of the orders due to their fat and protein content [26,27].

#### 3.1.2. Proteins

Protein content is one reason why edible insects are considered a viable alternative to other sources of animal and vegetable origin. In general, they have between 20 to 70% of quality and easily digestible proteins (digestibility has been estimated between 46 to 96%); these percentage variations will depend on the conditions described above. It has been determined that most orders contain all essential amino acids (representing 46 to 96% of the total amount of amino acids) and nonessential amino acids. Although some studies show low levels of methionine and tryptophan in some species, these values can be attributed to the methods used to analyze them and not necessarily because they are absent or at a low level [17,28].

In most cases, insects have high values of leucine, lysine, valine, threonine, phenylalanine, and histidine, while the rest of the essential and nonessential amino acids can equal or exceed recommended daily amounts. This content of essential amino acids can represent a valid nutritional option in some less-developed countries where the diet is usually of vegetable origin and deficient in some essential amino acids. As in African countries such as Angola and Papua New Guinea, which have a deficient intake mainly in lysine and leucine since their diet is based on tubers, the inclusion of termites of the genus *Macrotermes subhyalinus* and larvae of the *Rhynchophorus* family beetle can improve the diet when complemented with traditional tubers, which contain tryptophan and aromatic amino acids that are relatively deficient in the mentioned insects [11,14,28].

It is important to mention that the biological value (BV) of insect proteins is high compared to other protein sources. The BV of cricket (G. *assimilis*), moth (*Cirina forda*), grasshopper (*Melanoplus foedus*), and termite (*Macrotermes nigeriensis*) is between 85 to 93%, surpassing the BV of milk casein, which has a BV of 73%. Likewise, the protein efficiency ratios of these insects are comparable or higher than casein [29].

#### 3.1.3. Lipids

After proteins, fats are the most representative macronutrient in insects. The developmental stage of the insect must be taken into account to analyze whether the fat content is truly representative, since many species have a high fat content in their larval stage. This decreases when they have reached their maximum development, for example, the beetle *Rhynchophorus palmarum* L. (known as *chontacuro* in some South American countries). Its larval stage can have between 35 to 65% of fat in dry basis of its total composition, prevailing over protein. These values reverse when the insect becomes an adult. It must be noted that this particular insect is eaten in its larval stage; therefore, it must be analyzed for nutrient content appropriately, whether its consumption will be whole or its protein will be isolated in the form of flour [11].

A general range of lipids in insects is between 10 to 50% on a dry basis (taking into account the parameters of the development of the species, diet, and conditions of the species already mentioned). Importantly, in female insects, there is a higher fat content than in males. The lipid composition of insects is approximately 80% fatty acids and the rest corresponds to phospholipids and sterols, mainly cholesterol. On average, some species of bugs, cockroaches, termites, and some caterpillars have fat content values of 30%, while grasshoppers, crickets, and locusts can have a range of between 13.4 to 33.4% [27,30].

The fatty acid profile consists mainly of saturated fatty acids (SFA) and monounsaturated fatty acids (MUFAs) which can generally range from 30%. For example, in bees, ants, and wasp, up to 42% SFA is found. In termites and beetles, of which palmitic acid (C16:0) comprises 20% of the profile, an interesting content of myristic acid (C14:0) is also found (mainly in *T. molitor)*. MUFAs are also found in a range of 20% to 35% in all species except in black soldier fly larvae (7%), specifically oleic acid (C181 n-9) [30]. Finally, polyunsaturated fatty acids (PUFA) range, on average, 16% for flies and 39% for caterpillars of butterflies and moths, although some species of the orders Lepidoptera, Isoptera, Hymenoptera, and Orthoptera have been shown to have a higher concentration of PUFA than the normal average [27,31].

#### 3.1.4. Minerals and Vitamins

Another important nutritional aspect of insects are the minerals and vitamins they may contain. In terms of minerals, the presence of iron, calcium, phosphorus, potassium, zinc, copper, magnesium, and manganese stands out. There is a variable concentration of these minerals according to the order and species, and high concentrations can be found for a balanced diet. The caterpillar *Gonimbrasia belina* (Lepidoptera) has between 31–77 mg of iron per 100 g of dry matter and 14 mg of zinc per 100 g, while the grasshopper *L. migratoria* (Orthoptera) can have an iron concentration of 8 to 20 mg per 100 g of dry matter [28]. In general, insects, regardless of order and species, have a higher content of calcium, copper, manganese, and zinc when compared to the content of meat. Similarly, when calculating the nutritional quality index (INQ), it is noted that most minerals present in insects are well-balanced in terms of energy. For example, the adults of *Acheta domesticus* and *Gryllus bimaculatus* and the larva of *Gonimbrasia belina* can be used to supplement the diet of a daily ration of a feed that is deficient in K, P, Na, Mn, Cu, Fe, Zn, Mg, and Ca [32]. Studies have demonstrated that the bioavailability and solubility of minerals from edible insects is high. Data support that the iron bioavailability of beef sirloin is equal to that found in mealworms, grasshoppers, and buffalo worms [29].

On the other hand, the content of lipidic and water-soluble vitamins can be variable in each insect species, and it should also be mentioned that studies to further determine their content are lacking. It has been estimated that several insect species contain thiamine in a range of 0.1 to 7.7 mg of dry matter and riboflavin from 0.11 to 8.9 mg of dry matter, while vitamin B12 has been found, for example, in larvae of the yellow mealworm beetle *T. molitor* and in house cricket *Acheta domesticus* in concentrations of 0.47 and 5.4 μg of dry matter, respectively [28]. In general, insects are a good source of B vitamins, mainly vitamin B12. For this reason, insects can be an alternative for people who have plant-based diets. The house cricket can be incorporated into such a diet because, according to several studies, the species can have concentrations of 5.4 mg or 2.88 µg per 100 g of dry matter. These are values that correspond to the range of recommended daily intake for adults [33]. It has also been reported that cricket powder contains 10 times more vitamin B12 than beef, and that several species of insects may have higher vitamin A, C, and riboflavin content than beef, pork, and chicken (the comparison is between dry powder and traditional meat) [33,34].

#### 3.1.5. Fiber

The carbohydrate content of edible insects is represented by their fiber, which can be found in considerable amounts. The most common fiber that can be found is chitin mainly in their exoskeleton. Therefore, it will depend on the species and its developmental stage. In commercial farm species, a range of 12 to 137 mg per kg of dry matter, or 3 to 50 mg per kg of fresh weight, is found [28]. The chitin present in insects acts as cellulose in the human body since it cannot be digested. For this reason, it is usually called animal fiber; it is associated with the improvement in the immune system and it has also been associated with a reduction in allergic reactions in people and with good defense against parasitic infections [28,35].

The amount of fiber in some insect species has been estimated. A total of 8.5% of the weight of cricket powder is composed of dietary fiber from its chitinous exoskeleton, and it has been estimated that one of the insects with the highest fiber content is the African migratory locust (27%) while one of the insects with the lowest content is the Jamaican field cricket (8%) [34,36]. Chitin may contain nitrogen and bounded amino acids with the exoskeleton, that can alter the measurement of fiber content; it has been determined that, in beetles, crickets, and bees, chitin had between 9 to 32% of amino acids in its composition while the amount of nitrogen was negligible [29,37].

**Table 1:** Summary of the nutritional composition data of the main orders of edible insects commonly consumed worldwide.

Nutrient	Coleoptera (Beetles)	Diptera (Flies)	Hemiptera (True Bugs)	Hymenoptera (Ants–Bees–Wasps)	Isoptera (Termites)	Lepidoptera (Caterpillars– Butterflies–Moths)	Odonata (Dragonflies–Damselflies)	Orthoptera (Crickets–Grasshoppers– Locusts)	Blattodea (Cockroaches)
**kcal/100 g**	427–576	217–552	451–622	475- 555	-	349–777	431	336–485	-
**Protein** (%)	47–65	36–64	33–65	42–58	20–43	26–60	54–56	6–67	44–66
Amino acids (mg/g)									
Thr (23)	35.2	38.8	29.9	41.7	27.5	40	15.3	35.8	34.6
Trp (6)	10.1	28.3	10.3	10.3	14.3	11.2	19.4	8.1	6.0
Val (39)	53.8	46.9	44.3	60.5	73.3	54.1	16.5	50.3	53.8
His (15)	26.3	22.3	15.7	27	51.4	23.7	53.5	21.2	19.4
Ile (30)	45.6	32.6	31.5	47.8	51.1	40.4	14.2	39.6	29.9
Leu (59)	74.2	57.4	49.8	78.4	78.3	62.7	18.3	74.8	56.4
Lys (45)	50.6	52.9	28	53.8	54.2	57.7	22.5	53.9	48
Met (16)	16.2	27.2	21.7	23.8	7.5	22.1	4.1	19.3	29.8
Phe	47.1	50.6	34.4	47.5	43.8	46.3	8.6	46.6	30.6
Met + Cys (22)	31.9	36.6	32.2	30.5	26.2	34.7	-	29.8	41.4
Phe + Thy (30)	98.6	107.3	63.8	104.3	74	95.8	-	100.3	92.9
Arg	53.9	49.4	47.9	43.5	69.4	46.9	26.9	53.6	41.5
Cys	14.6	5.3	12.9	12.9	18.7	12.2	11	12.8	11.6
Tyr	55.7	56.7	38.7	55.3	30.2	-	9.5	61.5	62.3
Ala	69.5	58.9	26.4	72.3	-	48.9	20.7	77.4	56.6
Gly	55.2	45.1	16.4	81.3	-	43.8	21.4	54	58.7
Glu	123.7	98.6	23.7	134.3	-	103.4	40.5	94.5	99.7
Ser	42.6	60	10.3	38.2	-	48.4	15.7	41.9	41.9
Pro	64.1	27.8	-	66.7	-	44.9	43.2	53.9	65.0
**Lipids** (%)	30%	23%	34%	30%	27%	26%	20%	14%	23%
SFA (%)	2.4–42.2	2.9–8.8	41.5–57	14.5–34.6	0.1–31.9	19.4–39	-	2.34–44.5	28.3
MUFA (%)	3.4–48.1	2.6–4.7	1.2 7.3	1.96–70.2	0.2–1.87	4.4–50.8	-	3.1–38.4	10.3
PUFA (%)	0.8–64.5	1.1–3.6	43.8–53.4	15.7–63.6	0.3–65.3	1.7–62.1	-	0.95–58.4	78
**NFE** (%)	14	9	5	14	25	20	5	8	8
**Ash** (%)	6	10	4	3	3	5	9	4	5
**Fiber** (%)	14–18	9	5	11–14	25	14–21	5	8–27	8
**Vitamins (mg/kg)**									
Vitamin C	12–101	< 10	-	100.3	-	90	30	-	3.2–92
Thiamin	1.1–2.4	7.7	-	-	-	1–2–3.3	0.4	0.4	2
Riboflavin	8.1–11.2	16.2	-	-	-	9.4	34.1	34.1	16.6
Pantothenic acid	7–26.2	38.5	-	-	-	32.8	23	-	20.3
Niacin	35.6–46.5	71	-	-	-	2.6–33.6	38.4	3.8	4.4–29.5
Pyridoxine	3.55–8.1	2.3	-	-	-	1.74	2.3	-	2.13
B12 (ug/Kg)	5–9.9	56	-	-	-	0.1	54	5.4	23.7–193
Minerals (mg/kg-mg/g) *									
Iron	20.6	19.9–66.6	10.10	1.8–10	53.3 -116	2.1–77	78.9	0.7–18.7	60.3–64.8
Calcium	366	156–262	32	7.6–40	58.7–63.6	55–224.7	206	9.2–76.3	42.9–48.3
Phosphorus	2.2	2.1–2.6	-	100.5–730	-	203–666.7	500.3	-	125
Potassium	2.9	2.9–3.4	362.1	1150	-	1.9–1258	625.3	412.3	-
Zinc	52–54.3	49.5–56.2	10.10	10	8.1–10.8	11.1–25.9	5	8.5	7.1–13.8
Copper	6.1	3.6–8.3	-	1	-	1.2–3.3	1.34	1.5	-
Magnesium	193	620	74.55	50	-	254–402.2	73.13	0.1–87.2	0.2
Manganese	5.2–8.7	3.2–61.8	-	40	-	2.7–5.8	11.5	5	-

kcal/100 g: energy content. Table values in dry weight. Essential amino acid (in parentheses: recommended dietary allowance for adults): Thr: Threonine. Trp: Tryptophan. Val: Valine. His: Histidene. Ile: Isoleucine. Leu: Leucine. Lys: Lysine. Met: Methionine. Phe: Phenylalanine. Met + Cys: Methionine + cysteine. Phe + Thy: Phenylalanine + tyrosine. Nonessential amino acids: Arg: Arginine. Cys: Cysteine. Tyr: Tyrosine. Ala: Alanine. Gly: Glycine. Glu: Glutamic acid. Ser: Serine. Pro: Proline. A range was not established in the amino acids since, in many cases, only one or two values were found in the studies consulted. In some cases, several values were found and an average was applied. SFA: saturated fatty acid, MUFA: monounsaturated fatty acids, PUFA: polyunsaturated fatty acids. NFE: Nitrogen-free extract (indicative of soluble carbohydrates). * Minerals (mg/Kg: Coleoptera, Diptera and Hemiptera. mg/g: Hymenoptera, Isoptera, Lepidoptera, Odonata, Orthoptera and Blattodea). Values for energy content, fiber, protein, lipids, fatty acids, ash, carbohydrates and some vitamin and mineral values represent an average or range of various insects of each order. The values of fatty acids of Blattodea, some values of vitamins and minerals correspond to a single species of each order of insects in a different stage of development according to the consulted bibliography. (-): means undetectable or not found. Information adapted and modified from: Aguilar (2021); Akhtar and Isman (2018); Finke (2015); Fleta (2018); Guil-Guerrero et al. (2018); Hlongwane, Slotow, and Munyai (2020); Kouřimská and Adámková (2016); Maneechan and Prommi (2021); Meyer-Rochow, Gahukar, Ghosh, and Jung (2021); Ordoñez-Araque and Egas-Montenegro (2021); Prósper (2020); Rumpold and Schlüter (2015) and Séré et al. (2021) [4,14,15,17,27,28,31,38,39,40,41,42,43].

## 4. Edible Insects for Animals

### 4.1. Black Soldier Fly Larvae

The larvae of the black soldier fly (*Hermetia illucens*) are a popular source of protein as animal feed; it is an insect with an enormous reproductive capacity, rapid growth, and great capacity to process a range of by-products, and because of the high percentage of high-quality protein it produces. This type of fly does not transmit any disease to humans, animals, or plants [44]. The use of black soldier fly larvae or larval meals has been evaluated in trials with chickens, pigs, and tilapia, which, due to their qualities, allow easy incorporation and greater precision in the formulation of animal diets, providing crude protein and highly desirable lipids with medium chains (up to 76%), monounsaturated fatty acids (up to 32%), and polyunsaturated fatty acids (up to 23%) [45].

Black soldier larvae meal was found to be a suitable ingredient in growing pig diets, being especially valuable for its amino acid, lipid, and Ca content. However, its relative deficiency in methionine, cystine, and threonine necessitates the inclusion of these amino acids for the preparation of balanced diets. In addition, this meal also favors good growth of poultry and could totally or partially substitute fish meal in fish diets [46]. 

### 4.2. Mealworm Larvae

The mealworm larvae are especially used for feeding fish such as rainbow trout (*Oncorhynchus mykiss*), mahi-mahi (*Sparus aurata*), and tilapia (*Oreochromis niloticus* L.). *Tenebrio molitor,* which is the scientific name of the yellow or mealworm, is a coleopteran that provides good results at the nutritional level, as it has excellent nutritional characteristics and among them stands out its excellent content of easily digestible protein, as well as the presence of monounsaturated and polyunsaturated fatty acids (especially oleic and linoleic), minerals, and vitamins, and even the production of bioactive peptides has been reported [47].

This beetle feeds on grains, flours, and their by-products; its larvae readily breed on plant and animal products of low nutritional value. They are commercially produced to be used as pet food (birds and reptiles) or fishing baits. They are easy to breed and rear and have a stable protein content regardless of their diets, implying that *T. molitor* larvae can be stably produced. For this reason, they have been produced industrially as food for pets, zoo animals, and even for production animals, such as fish, pigs, and poultry [48,49].

### 4.3. Giant Mealworm and Small Mealworm

Giant mealworms (*Zophobas atratus* Fab.) and small mealworms (*Alphitobius diaperinus* Panzer) are grown on diets composed of organic by-products originating from brewing, bread/biscuit baking, potato processing, and bioethanol production [49]. Giant mealworms (Coleoptera: Tenebrionidae) are a large Neotropical beetle and also a well-known food resource for small pets, but are several times larger than a mealworm. They have been used as a protein source for small pets such as birds, reptiles, and small mammals [50].

Three species of edible larvae of the beetle family Tenebrionidae, better known as mealworms, are currently produced commercially: the yellow mealworm, the giant mealworm, and the small mealworm, these insects are commonly produced in mixed-grain diets. Recently, separate production lines have been established in the Netherlands to facilitate the production of *T. molitor* and *A. diaperinus* for human and animal consumption [51].

### 4.4. Locusts, Grasshoppers, and Crickets

Locusts, grasshoppers (mainly Acrididae and Pyrgomorphidae), crickets (Gryllidae), and leafhoppers (Tettigoniidae) are insects of the order Orthoptera. Orthoptera, like other insects, are highly nutritious and contain high amounts of protein. Several grasshoppers, leafhoppers, and cricket species are already used for raising pets and zoo animals and have been investigated for livestock feed [52].

In particular, the availability of large quantities of dead locusts resulting from locust outbreaks makes them a good potential feed for livestock, especially poultry. In the Philippines, free-range chickens fed with grasshoppers have been reported to have a more palatable taste and command a higher market price than those fed with conventional commercial feeds. More recent studies have tried to replace a portion of the fish meal with lobster and grasshopper meal and found that such partial substitution is generally adequate [53,54].

## 5. Possible Risks to Be Taken into Account When Consuming Insects

An important aspect to consider for the production and consumption of farm-produced insects for humans and animals is the potential health risks that may be present. These risks are those also found in conventional food: antinutrients, allergens, heavy metals, and chemical and microbiological hazards. The occurrence of these risks is determined by feeding during rearing, harvesting conditions, transport, drying methods, storage, and distribution, as well as the species and stage of development that will be destined for consumption. As for microbiological hazards, microorganisms can appear in the digestive tract or on the surface. Several studies have isolated some pathogenic bacteria in the digestive tract of different insect species: *Salmonella* spp., *Campylobacter* spp., *E. coli* O157:H7, *Staphylococcus aureus*, *Listeria* spp., *Clostridium* spp., and *Bacillus cereus*, all spore-forming bacteria that can survive heat treatments, have also been found [55,56].

As for the presence of molds, *Aspergillus, Penicillium, Fusarium,* and *Beauveria bassiana* have been found in insects. These have the ability to generate mycotoxins. The presence of aflatoxins, eniantin A and A1, and beauvericin has been detected in considerable concentrations in some insect samples. To a lesser extent, the presence of parasites has been found in insects as vectors such as *Toxoplasma gondii, Entamoeba histolytica, Giardia lamblia, Dicrocoelium dendriticum, Phaneropsolus bonnei, Prosthodendrium molenkampi,* and *Hymenolepsis diminuta*. These are the main parasites found in insects in the wild such as cockroaches, flies, dragonflies, yellow mealworm, and ants [55]. As for the allergies that can appear after the consumption of insects, we can highlight species that have considerable amounts of chitin since humans have a deficiency of the enzyme chitinase. This deficiency can cause allergic reactions in the same way that people are allergic to seafood such as shrimp [28].

The possible presence of some antinutrients in insects should be considered since these molecules can alter the bioavailability of proteins and minerals. The presence of oxalates, alkaloids, and saponins has been reported in some insect species (*H. whellani* and *Macrotermes facilger*) [29]. Finally, another risk that can appear in the consumption of edible insects is heavy metals, mainly arsenic, mercury, cadmium, and lead. These can accumulate in the body of insects throughout their life due to different factors such as species, developmental stage, and feed substrate. The presence of arsenic and cadmium has been found in yellow mealworm larvae, Bombay locust, scarab beetle, black soldier flies, house cricket, and mulberry silkworm [57].

## 6. Environmental Impact of Edible Insects

As already mentioned, the use of insects for human and animal consumption is an option to mitigate to a certain extent the current problems of climate change and the environmental impact that the planet is facing today. The demand for meat products increases every year (an increase of 75% is expected by the year 2050) and with this, environmental problems are also expected to increase. It is estimated that meat represents around 15% of the total energy in diets of people. In addition, consuming animal-based protein reduces the supply of plant-based foods, such as cereals as they are used to feed cattle, pigs, and poultry (a third of world cereal production is used for animal feed). On the other hand, we must highlight the effects of the emission of greenhouse gases into the atmosphere generated by animals that are intended for human consumption [58].

Cattle are responsible for 78% of these emissions. The main gases generated are me-thane (43%), nitrous oxide (29%), and carbon dioxide (27%), which are formed from enteric fermentation, manure, changes in land use, and fossil fuels. In terms of land use and water needs, insects are presented as an alternative to minimize the use of resources on a large scale, since they need much less soil and water per unit of protein provided. This protein will have a greater feed conversion efficiency; for example, on average, edible insect species have the ability to convert 2 kg of feed into 1 kg of insect mass, while cattle need 8 kg to achieve 1 kg of body weight gain [3,58].

The water footprint is important to determine if there is excessive use of water in industry. To calculate its use in livestock production, water used in the feed, to drink, and in service water consumption is analyzed, and it has been determined that the average annual water footprint (m^3^/year/animal) is 631 for beef cattle, 521 for pigs, 26 for broiler chickens, and 0.003 for mealworms. It is important to mention that the comparison of the need for land, water, food, fossil fuel expenditure, and resources for food production between insects and traditional animals must be made considering an edible fraction, since, unlike beef, chicken, and pork where there is a lot of waste (bones, viscera, and skin), the edible fraction of insects is around 80% to 90%. For all these reasons, it is essential to look for alternatives to the consumption of conventional foods that come from an industry that is committed to increasing climate change [59,60,61]. Table 2 shows the environmental impact on different parameters of insects and other traditional species for human consumption.

## 7. Conclusions

It is important to generate strategies that allow sustainable food production. In this case, insects as a whole represent a large animal biomass on the planet, and in every ecosystem, they are an important source of protein. The use of land, water, greenhouse gas emissions, and energy are generally lower for insect production compared to other animal and vegetable protein sources.

Insects have an interesting nutritional profile with regard to their content of macro- and micronutrients. The type of insect and stage of development determine that which is best used for animal feed. So, depending on the order in which they belong, insects can meet the nutritional requirements of animals and can replace traditional feed. With the development of technology with which these aspects are analyzed, it will be possible to create insect farms where insects with good nutritional profiles are raised.

Edible insects produced on farms have the same risks as conventional foods. For this reason, all safety and quality policies must be followed to avoid contamination by microorganisms, chemical contaminants, accumulation of heavy metals, and the presence of antinutrients and allergens. Emphasis must be placed on legislation to create production protocols and thus avoid possible health risks to consumers.

## Figures and Tables

**Table 2:** Main parameters that generate environmental impact of edible species.

Animal Category	Land (Land Use m^2^ for 1 kg of Protein)	Water Required (L/g of Protein)	Feed (kg/kg of Live Weight)	A-GHG Production (g/kg Mass Gain)
Insects	20	23	2	1
Beef cattle	145–255	112	10	2850
Broiler chicken	40–55	34	3	300
Pork	45–70	57	5	1300

A-GHG: Average Greenhouse Gas. Water required data for insects are referenced for mealworms. Data obtained from the following researchers: Guiné, Correia, Coelho, and Costa (2021); Miglietta, De Leo, Ruberti, and Massari (2015) and Sogari (2015) [3,59,61].

## Data Availability

The data presented in this study are available on request from the corresponding author.
